# Supplementary material for: The Construction and Exploration of a Comprehensive MicroRNA Centered Regulatory Network in Foxtail Millet (Setaria italica L.)
Source: Front Plant Sci. 2022 May 6;13:848474. doi: 10.3389/fpls.2022.848474 (PMC9121102; doi:10.3389/fpls.2022.848474)
Supplement: Supplementary file 4 [file Data_Sheet_4.zip › Sit-miRN3000_Seita.5G104000.1_700_TPlot.pdf]

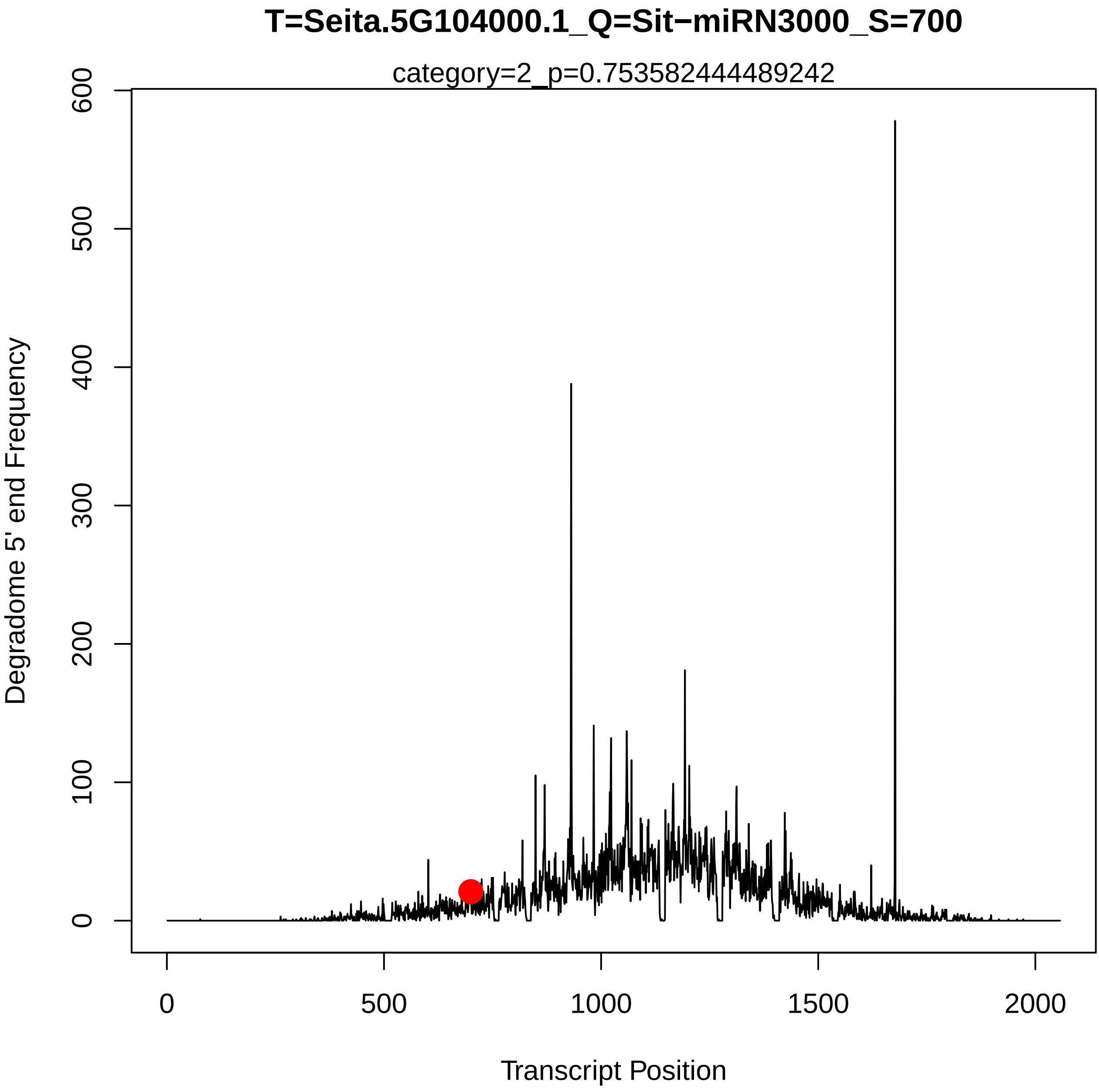

T=Seita.5G104000.1_Q=Sit−miRN3000_S=700
category=2_p=0.753582444489242
Degradome 5' end Frequency
0
100
200
300
400
500
600
0
500
1000
1500
2000
Transcript Position
